# Imatinib Mesylate and Sirolimus Inhibit Vascular Fibroproliferative Remodeling in Pulmonary Vein Stenosis

**DOI:** 10.3390/children13091280

**Published:** 2026-09-21

**Authors:** Julia Gaal, Sophie C. Doettl, Kerstin Saraci, Cindy Zajac, Alexa von Mueffling, Peter E. Hammer, Magnus von Piechowski, Verena Gebert, Andrei-Antonio M. Caracioni, Daniel Diaz-Gil, Viktoria Weixler, Pedro J. Del Nido, Kathy Jenkins, Ingeborg Friehs

**Affiliations:** 1 Department of Cardiac Surgery, Boston Children’s Hospital, Boston, MA 02115, USA; julia.gaal@charite.de (J.G.); sophie.doettl@jku.at (S.C.D.); kerstin.saraci@campus.lmu.de (K.S.); alexa.vonmueffling@childrens.harvard.edu (A.v.M.); peter.hammer@childrens.harvard.edu (P.E.H.); magnus-paul.von-piechowski@charite.de (M.v.P.); andrei-antonio.caracioni@childrens.harvard.edu (A.-A.M.C.); pedro.delnido@cardio.chboston.org (P.J.D.N.); 2 Department of Congenital Heart Surgery-Pediatric Heart Surgery, German Heart Center, Charité—Universitätsmedizin Berlin, 13353 Berlin, Germany; viktoria.weixler@dhzc-charite.de; 3 Department of Surgery, Harvard Medical School, Boston, MA 02115, USA; 4 Department of Cardiology, Boston Children’s Hospital, Boston, MA 02115, USA; daniel.diaz-gil@childrens.harvard.edu (D.D.-G.); kathy.jenkins@cardio.chboston.org (K.J.); 5 Department of Pediatrics, Harvard Medical School, Boston, MA 02115, USA

**Keywords:** pulmonary vein stenosis, fibroproliferative remodeling, endothelial-to-mesenchymal transition, wall shear stress, anatomical focused repair, congenital heart disease, imatinib mesylate, sirolimus

## Abstract

**Highlights:**

**What are the main findings?**

- In three patients with pulmonary vein stenosis (PVS), fibroproliferative remodeling was observed in pulmonary veins resected during surgical anatomical repair, which exhibited increased wall shear stress (WSS). The cellular substrate underlying this process appeared to be driven by endothelial-to-mesenchymal transition (EndMT) of endothelial cells (ECs).
- In vitro studies demonstrated that clinically relevant concentrations of imatinib mesylate and sirolimus significantly inhibited EndMT in ECs derived from sites of PVS.

**What is the implication of the main finding?**

- Fibroproliferative ECs undergoing EndMT are likely the cellular drivers of PVS and may be amenable to targeted therapy with imatinib mesylate and sirolimus.

**Abstract:**

**Background:** Pulmonary vein stenosis (PVS) is a progressive disease characterized by neointimal hyperplasia with poor outcomes. Clinical observations show that adjunctive therapy with imatinib mesylate and sirolimus is associated with improved survival and reduced rates of reintervention. In this study, we aimed to investigate the cellular mechanisms underlying PVS development and progression and to elucidate how these targeted therapies modulate these processes. **Methods:** We retrospectively analyzed cardiac catheterization and computed tomography, and lung scan data from three patients with PVS to estimate wall shear stress (WSS) at multiple time points. Tissue specimens resected during anatomical repair surgery were analyzed for tissue composition and fibrosis using hematoxylin and eosin staining, Masson’s trichrome staining, and flow cytometric analysis of endothelial cell (ECs) and fibroblast markers to determine endothelial-to-mesenchymal transition (EndMT). The safety and efficacy of imatinib mesylate and sirolimus were evaluated in healthy and PVS-derived ECs using cellular viability and proliferation assays, and by assessing fibrotic marker expression indicative of EndMT. **Results:** Patients exhibited severely elevated WSS estimates at multiple time points throughout disease progression. Disproportionate fibrotic remodeling driven by ECs transitioning into mesenchymal cells through EndMT was the cellular substrate of resected tissue in PVS. Targeted therapy with clinically relevant concentrations of imatinib mesylate and sirolimus was safe and significantly inhibited fibroproliferation and EndMT. At follow-up, all patients were alive and free of surgical and stent-based reintervention after anatomic repair surgery and adjunctive imatinib mesylate and sirolimus therapy. **Conclusions:** Fibroproliferative ECs undergoing EndMT are likely the cellular substrate underlying PVS development and progression. A multimodal approach consisting of anatomically focused surgical repair with adjunctive imatinib mesylate and sirolimus therapy may attenuate fibroproliferative remodeling.

## 1. Introduction

Pulmonary vein stenosis (PVS) is a rare obstructive disease of the pulmonary veins that is characterized by intraluminal neointimal hyperplasia. It can occur as a primary disease, in the context of congenital heart defects (CHDs), or secondary to localized interventions [1,2]. The disease trajectory frequently leads toward complications such as pulmonary hypertension (PH) and right ventricular (RV) heart failure, resulting in poor outcomes [3,4,5,6,7]. Aggressive surgical or catheter-based therapies are repetitive and often fail to control disease progression and recurrence [6,8,9]. Despite promising advances, clinical observations suggest that attempts to restore a more physiological flow profile alone are insufficient to achieve disease control [9]. This underlines the need to better understand the underlying cellular mechanisms. The typical neointimal hyperplasia and disproportionate extracellular matrix in the affected veins may result from fibroproliferative vascular processes [10,11,12,13]. Endothelial-to-mesenchymal transition (EndMT), the transition from endothelial cells (ECs) into mesenchymal cells, can contribute to fibroproliferative vascular diseases in the cardiovascular system and is modulated by fluid shear stress [14]. Recently, a case study suggested that elevated wall shear stress (WSS) may contribute to the disease process in PVS [15]. However, given the limited success of current management strategies, our knowledge of the underlying disease mechanisms in PVS development and progression remains incomplete.

This points out the need for adjunctive therapies in addition to surgical and catheter-based interventions. More recently, two pharmacologic agents targeting vascular cell proliferation have been introduced in the treatment of patients with PVS: the tyrosine kinase inhibitor imatinib mesylate and the mammalian target of rapamycin (mTOR) inhibitor sirolimus. Both drugs have demonstrated beneficial effects in this population. Although neither therapy eliminates disease recurrence, both have been associated with improved overall survival and/or a reduced need for catheter-based and surgical reinterventions [16,17,18,19,20]. In addition to anti-proliferative effects, imatinib mesylate and sirolimus have demonstrated anti-fibrotic effects on EndMT [21,22]. Considering these promising outcomes, further investigation of their underlying mechanisms of action is warranted.

We aimed to investigate the cellular mechanisms underlying PVS development and progression and to elucidate how imatinib mesylate and sirolimus exert their beneficial effects in patients with PVS by altering these mechanisms.

## 2. Materials and Methods

### 2.1. Patients

For the clinical case presentation, we included three patients who underwent anatomic surgical repair for PVS at Boston Children’s Hospital from June 2024 to August 2025. These patients had resected tissue specimens available for histological analysis. Three preoperative cardiac CT scans were also performed. Clinical follow-up was available through July 2026.

### 2.2. Calculation of In Vivo Wall Shear Stress

WSS estimates were calculated based on a retrospective analysis of serial cardiac catheterization, cardiac computed tomography (CT), and lung scan data, as previously published [15]. Briefly, for each vein, the site with the smallest vessel cross-sectional area was identified at the ostium, near the confluence with the atrium. At this location, both the maximum and minimum vessel diameters were measured. WSS was then estimated for each diameter, assuming steady flow. The maximum diameter yielded the minimum WSS, whereas the minimum diameter yielded the maximum WSS. Diagnostic cardiac CT was used to assess local disease progression in the context of worsening clinical symptoms.

### 2.3. Tissue Characterization

Representative tissue samples were analyzed for overall morphology using hematoxylin and eosin (H&E) staining. The collagen content (fibrosis) was assessed using Masson’s trichrome (MT) staining. Tissue sections were imaged using an Axio Observer Z1 (Carl Zeiss, Oberkochen, Germany) inverted microscope with ZEN3.6 (blue edition) software at 20× magnification. Flow cytometry was conducted as previously described in detail [23]. Briefly, to quantify EndMT, flow cytometry with the cell surface marker PE-conjugated CD31 (1:100, #303106, BioLegend, San Diego, CA, USA) and APC-conjugated fibroblast markers (1:50, #130-123-827, Miltenyi Biotec, Gaithersburg, MD, USA) was performed on single cells. Isotype controls were used to assess nonspecific binding.

### 2.4. Isolation and Maintenance of Human ECs

Tissue was resected at the venoatrial junction from patients with pulmonary vein stenosis and from healthy controls without pulmonary vein stenosis. ECs were isolated and cultured as we have previously described in detail [23,24]. Briefly, tissue samples obtained from the operating room were minced, enzymatically digested, and cultured in endothelial cell growth medium-2 (ECGM-2; #CC-3162, Lonza Bioscience, Walkersville, MD, USA) supplemented with components required for EC growth, as provided by the manufacturer. Additionally, the medium was supplemented with penicillin-streptomycin-glutamine (250 mg/L, #15140122, Gibco, Waltham, MA, USA) and the transforming growth factor beta 1 (TGF-β1) inhibitor SB431542 (#130-106-543, Miltenyi Biotec, Gaithersburg, MD, USA) to prevent cellular dedifferentiation during culture. Healthy control ECs (HECs) were obtained from cardiovascular-healthy pediatric donors postmortem. The pulmonary veins were cut open to expose the luminal surface, placed lumen-side down in digestion buffer, and processed using the same isolation protocol. Prior to experiments, SB431542 was washed out for 24 h. For experimental conditions, imatinib mesylate (#HY-50946, MedChemExpress, Monmouth Junction, NJ, USA), sirolimus (#HY-10219, MedChemExpress, Monmouth Junction, NJ, USA), dimethyl sulfoxide (DMSO, #276855, Sigma-Aldrich, St. Louis, MO, USA) or no treatment were used.

### 2.5. MTT Assay

First, 20,000 cells were plated in a 96-well plate and allowed to adhere for 24 h in EGM-2. After 24 h, the medium was then replaced with one of the following experimental conditions: imatinib mesylate (0.5, 3, and 10 µM), sirolimus (0.5, 3, and 10 nM), vehicle control (0.1% DMSO), or no treatment (native control). Cells were cultured for 72 h, with one medium change after 48 h. Subsequently, the culture medium was removed and then replaced with 100 µL of fresh EGM-2. Then, 10 µL of a 12 mM MTT (3-(4,5-dimethylthiazol-2-yl)-2,5-diphenyltetrazolium bromide; #M6494, Invitrogen, Carlsbad, CA, USA) stock solution was added. Next, the plate was incubated at 37 °C for 3.5 h. After incubation, all but 25 µL of the medium was removed, and 50 µL of DMSO was added. The solution was thoroughly mixed by pipetting and then incubated again at 37 °C for 10 min. Absorbance was measured at 540 nm using a spectrophotometer.

### 2.6. Apoptosis Assay

First, 300,000 cells were seeded in a 60.1 cm^2^ Petri dish and allowed to adhere for 24 h in EGM-2. After 24 h, the medium was then replaced with one of the following experimental conditions: untreated control (vehicle), imatinib mesylate (10 µM), or sirolimus (10 nM). Cells were cultured for 72 h, and the medium was changed after 48 h. Cells were dissociated into single-cell suspensions using 0.05% trypsin (#25300054, Gibco, Waltham, MA, USA) and a trypsin neutralizer solution (#R002100, Gibco, Waltham, MA, USA), followed by washing with fluorescence-activated cell sorting (FACS) buffer consisting of phosphate-buffered saline (PBS, #10010023, Gibco, Waltham, MA, USA) supplemented with 1% bovine serum albumin (BSA, #A4737, Sigma-Aldrich, St. Louis, MO, USA) and 0.2 mM EDTA (#15575020, Invitrogen, Carlsbad, CA, USA). The APC Annexin V Apoptosis Detection Kit with Propidium Iodide (PI) (#640932, BioLegend, San Diego, CA, USA) was used as described by the manufacturer. First, cell pellets were resuspended in 100 µL of Annexin V binding buffer. Second, 5 µL of fluorochrome-conjugated Annexin V and 5 µL of viability dye PI were added. The suspension was vortexed and incubated for 15 min at room temperature (RT) in the dark. Then, 400 µL of Annexin V binding buffer was added, and samples were analyzed immediately by flow cytometry. A minimum of 30,000 single cells were acquired per sample. Gates were defined based on the untreated control and applied to all treatment conditions.

### 2.7. Proliferation Assay

Cells were cultured and treated as described above. Cell proliferation was assessed using the Click-iT Plus EdU Alexa Fluor 647 Flow Cytometry Assay Kit (#C10634, Invitrogen, Carlsbad, CA, USA) according to the manufacturer’s instructions. Briefly, cells were labeled with 10 µM 5-ethynyl-2′-deoxyuridine (EdU), then harvested, washed, and fixed and permeabilized. Following incubation with the EdU reaction cocktail, incorporated EdU, marking newly synthesized deoxyribonucleic acid (DNA), was detected by flow cytometry. A minimum of 30,000 single cells were acquired per sample. Gates were set according to the untreated control and applied across all experimental conditions.

### 2.8. Immunofluorescence Staining

Cells were seeded on coverslips in a 24-well plate at a density of 50,000 cells per well and allowed to adhere for 24 h. The following day, cells were treated with one of the following experimental conditions: 3 µM imatinib mesylate, 3 nM sirolimus, or left untreated (vehicle). Each treatment was applied under two conditions: stimulation with 100 ng/mL human TGF-β1 protein (#240-B-010/CF, R&D Systems, Minneapolis, MN, USA), a well-established inducer of EndMT (disease model), or no stimulation (control).

After 72 h, cells were fixed with 4% paraformaldehyde (#J19943.K2, Thermo Fisher, Waltham, MA, USA) and blocked for non-specific binding using washing buffer (PBS containing 5% normal goat serum (NGS, #31872, Invitrogen, Carlsbad, CA, USA) and 0.3% Triton X-100 (#X100, Sigma-Aldrich, St. Louis, MO, USA)) at RT for 45 min. Primary antibodies diluted in incubation buffer (PBS with 1% bovine serum albumin (BSA), 1% NGS, and 0.3% Triton X-100) were applied overnight at 2–8 °C. Endothelial cells were stained using anti-CD144 (cluster of differentiation 144; 1:100, #14-1449-82, Invitrogen, Carlsbad, CA, USA), and mesenchymal cells were stained using anti-α-smooth muscle actin (αSMA; 1:100, #ab124964, Abcam, Waltham, MA, USA). The following day, cells were washed twice and incubated with secondary antibodies (anti-mouse IgG Alexa Fluor 647, 1:200, #A32728, Invitrogen, Carlsbad, CA, USA; and anti-rabbit IgG Alexa Fluor 488, 1:400, #A-11034, Invitrogen, Carlsbad, CA, USA) in dilution buffer at RT in the dark for 1 h. After two additional washes, cells were incubated with 4′,6-diamidino-2-phenylindole (DAPI; 1:5000, #D1306, Invitrogen, Carlsbad, CA, USA) at RT for 5 min, then washed with PBS and distilled water. Stained slides were stored at 2–8 °C in the dark until imaging.

Images were acquired using an Axio Observer Z1 inverted microscope (Carl Zeiss, Oberkochen, Germany) with ZEN3.6 (blue edition) software and a 10X objective. For each experiment, five random fields were captured. Cell detection was based on nuclear staining, and relative marker expression was semi-quantified within the cytoplasmic compartment using mean fluorescence intensity in the Alexa Fluor 488 and Alexa Fluor 647 channels. Mean fluorescence intensity was defined as the average pixel intensity within segmented cells and quantified using QuPath software (version 0.6.0). Data were averaged per 100 CD144+ ECs and normalized to the untreated control.

### 2.9. Statistical Analysis and Reproducibility

Clinical case presentations were descriptively summarized. All experiments were independently repeated in biological triplicates or more. Data derived from cell experiments were assessed for normality using the Shapiro–Wilk test and Q-Q plots. Data derived from HECs were analyzed using ordinary one-way ANOVA followed by Tukey’s post hoc test. Data derived from PVS-ECs (patient samples) were analyzed using repeated-measures one-way ANOVA followed by Tukey’s post hoc test. This accounted for individual-patient-level variance (matched samples). Data are shown as mean with standard deviation (SD). Error bars in graphs represent the standard error of the mean (SEM). All statistical analyses were performed using GraphPad Prism version 10 (GraphPad Software Inc., San Diego, CA, USA). Statistical significance was defined as *p* < 0.05.

## 3. Results

### 3.1. Clinical Case Presentation

#### 3.1.1. Case 1

Patient 1, a male, was born at full term and was diagnosed directly postnatally in the setting of respiratory distress with obstructive total anomalous pulmonary venous return (TAPVR) with drainage of all pulmonary veins into the coronary sinus, an atrial septal defect (ASD), and a patent foramen ovale (PFO). Progressive tachypnea and growth faltering then prompted initial surgical repair at the age of 0.9 months consisting of unroofing of the coronary sinus, enlargement of the pulmonary venous confluence to the LA, and fenestrated ASD closure. At the age of 2.7 months, his course was complicated by progressive bilateral PVS resulting in tachypnea and growth faltering. He presented with PVS of his left common pulmonary vein, right upper pulmonary vein (RUPV), and right lower pulmonary vein (RLPV). Cardiac CT showed moderate-to-severe bilateral PVS with significant interval progression. The common left pulmonary vein, RUPV, and RLPV were moderately to severely narrowed at their entry points into the LA with good-sized dilated distal vessels. In addition, cardiac catheterization demonstrated severe RV hypertension with suprasystemic pressures (RV/systemic pressure ratio 1.63) and severe PAH (mean pulmonary arterial pressure (mPAP) 75 mmHg). The patient had severe proximal stenosis with pulmonary vein gradients of 26 mmHg in the left common PV, 16 mmHg in the RUPV, and 20 mmHg in the RLPV. However, balloon dilation (BD) reduced the gradients to 4 mmHg, 3 mmHg, and 6 mmHg, respectively, with subsequent improvement in RV pressures to subsystemic levels (RV/systemic ratio 0.84). Given the severe PH caused by the pulmonary venous obstruction (PVO), the patient underwent anatomic repair surgery at 3.0 months of age using atrial rotational flap augmentation, left atrial augmentation with a thin pulmonary homograft patch, and fenestrated ASD closure. Mixed-tissue samples from the left common PV, RUPV, and RLPV were resected intraoperatively and sent for detailed downstream analysis for this study. The total follow-up period since anatomic repair surgery was 17.4 months.

The patient was directly started on imatinib mesylate therapy following anatomic repair surgery. Then, he underwent a total of seven catheter-based reinterventions for recurrent PVS, with serial BD of the LLPV, LUPV, RUPV, and RLPV at 4.6, 5.9, 8.0, 10.3, 12.8, 17.0, and 19.9 months of age (1.6, 3.0, 5.0, 7.3, 9.8, 14.0, and 17.0 months postoperatively). At 5.9 months of age (3.0 months postoperatively), he demonstrated recurrent severe proximal narrowing of all pulmonary veins with gradients of 13–16 mmHg, which were reduced to residual gradients of 1–4 mmHg following BD. He also had elevated right-sided filling pressures, with severely elevated RV pressures at systemic levels (RV/systemic pressure ratio 0.94) and severely elevated mPAP (41 mmHg). Following this reintervention, he was additionally initiated on sirolimus as antiproliferative therapy. At 8.0 months of age (5.0 months postoperatively), cardiac CT demonstrated progressive left-sided PVO. The LUPV had near-ostial atresia, and the LLPV had severe ostial narrowing. However, stenosis was less pronounced on the right side, with mild ostial narrowing of the RUPV and RLPV. At 12.8 months of age (9.8 months postoperatively), dapagliflozin propanediol was additionally introduced as adjunct therapy together with established antiproliferative treatment. His last catheter-based reintervention at 19.9 months of age (17.0 months postoperatively) demonstrated mildly elevated right-sided filling pressures (RAP 10 mmHg), severely elevated RV pressures at subsystemic levels (RV/systemic pressure ratio 0.67), and mildly to moderately elevated mPAP (26–30 mmHg). The LUPV showed recurrent ostial stenosis with additional long-segment narrowing and a reduction in pressure gradient from 12 mmHg to 0–1 mmHg after BD. The LLPV showed recurrent moderate-to-severe stenosis, with a gradient of 14 mmHg and 4 mmHg after BD. The RUPV had recurrent ostial stenosis, with a gradient of 6 mmHg that decreased to 2 mmHg after BD. The RLPV also showed mild-to-moderate recurrent ostial stenosis, with a gradient of 8 mmHg that decreased to 2 mmHg following BD. At the most recent follow-up, echocardiography demonstrated some accelerated flow with mean gradients of 2–5 mmHg in the diseased veins and signs of elevated right ventricular systolic pressure. The patient continued imatinib mesylate and sirolimus therapy.

#### 3.1.2. Case 2

Patient 2, a full-term male, was diagnosed directly postnatally with TAPVR (connection of all pulmonary veins to the right atrium), a leftward malposed atrial septum, a left superior vena cava to the coronary sinus, and a stretched PFO. Diagnosis was made in the setting of respiratory distress. Growth faltering and progressive turbulence of the pulmonary veins prompted initial surgical intervention at 0.5 months of age, consisting of baffling the pulmonary veins to the LA and unroofing the left-sided pulmonary veins, and ASD patch closure. The postoperative course was complicated by progressive left-sided pulmonary vein disease as evidenced by LUPV ostial atresia and mild stenosis of the LLPV ostium. He was subsequently started on imatinib mesylate as anti-fibroproliferative therapy. With disease progression, he required five catheter-based reinterventions at 2.5, 4.6, 6.4, 8.7, and 10.7 months of age. This included initial stenting of the LUPV and BD of the LLPV. Four additional BDs of the LUPV and two BDs of the LLPV followed. Following the first episode of in-stent stenosis, sirolimus therapy was additionally initiated for an eight-week course. Cardiac CT demonstrated PVS involving the LLPV and LUPV, with intimal proliferation within the LUPV stent. The LLPV appeared flattened along its course anterior to the descending aorta and showed narrowing proximal to its entry into the LA. Cardiac catheterization showed mild-to-moderate LUPV in-stent stenosis with distal obstruction, and LLPV compression between the heart and aorta with ostial tissue obstruction. BD of the LUPV and LLPV reduced gradients to 2 mmHg with improved flow. The RUPV and RLPV were unobstructed. At the age of 12.4 months, the patient presented again to our institution to be considered for left-sided pulmonary vein repair due to persistent PVS in the setting of no improvement in the frequency of catheter-based reinterventions. He underwent anatomic repair surgery of the obstructive left-sided pulmonary vein through stent removal of the LUPV, flap augmentation, and LUPV-LA reanastomosis. Additionally, the PFO and ASD were closed. We obtained resected tissue from the LUPV for downstream analysis for this study. The total follow-up period since anatomic repair surgery was 12.7 months.

The patient underwent two catheter-based reinterventions at 14.9 and 21.8 months of age (2.6 and 9.4 months after his anatomic repair surgery, respectively). Cardiac CT demonstrated persistent PVS involving the LLPV, LUPV, and RMPV. The LUPV showed mild compression along its course anterior to the left mainstem bronchus. Overall vein patency was significantly improved, without evidence of ostial stenosis. The LLPV was compressed along its course anterior to the descending aorta. Mild stenosis of the RMPV was present just proximal to its drainage into the RLPV. The RLPV and RUPV appeared unobstructed. The findings from this most recent catheter-based reintervention showed mildly elevated right-sided filling pressures (RAP 7 mmHg), mildly elevated RV pressures at subsystemic levels (RV/systemic pressure ratio 0.39), and normal PA pressures (17–19 mmHg). The RUPV and RLPV were both unobstructed with negligible 1–2 mmHg gradients. Following anatomic repair, the LUPV and lingular PV entered the LA separately. However, both were nearly atretic and underwent serial BDs to achieve residual gradients of 3–4 mmHg. The LLPV had a stable 3 mmHg gradient. This was due to extrinsic compression by the aorta as it courses posterior to the LA, rather than a true intraluminal PVO, as further demonstrated by intravascular ultrasound. At the most recent follow-up, echocardiography showed unobstructed flow in both the left and right pulmonary veins. The RV function was good, and there was no evidence of RV hypertension. The patient remained on imatinib mesylate therapy.

#### 3.1.3. Case 3

Patient 3, a female, was a referral from an outside hospital with a history of partially anomalous pulmonary venous return (PAPVR) (right-sided pulmonary veins draining into the inferior vena cava, scimitar syndrome), left-sided PVS, and an ASD. She underwent initial surgical repair at 11.0 months of age. The surgery included baffling of the scimitar vein to the LA, intraoperative stenting of the LUPV and LLPV, and placement of a fenestrated ASD patch. Her course was severely complicated by Scimitar baffle atresia and recurrent multivessel PVS, resulting in management challenges. A total of 17 BDs and stenting procedures involving the LUPV, LLPV, RUPV, RMPV, and RLPV were performed. She underwent four surgical reinterventions, including Scimitar baffle revision, LLPV stent dilation, and enlargement of the ASD fenestration at 20.6 months of age; Scimitar baffle revision and dilation of the anomalous RLPV at 24.1 months of age; and, at 9.6 years of age, unroofing of the RUPV, removal of pulmonary vein stents, atrial septectomy, and atrial septal reconstruction. Regardless, her pulmonary vein disease progressed, and in the setting of ongoing neoproliferation, imatinib mesylate therapy was initiated at 10.7 years of age but discontinued after three months because of severe lower-extremity pain. Cardiac catheterization demonstrated that the RUPV drained into the RLPV via interlobar collaterals. The RLPV consisted of two closely adjacent vessels draining through a common ostium, which was re-stented and balloon-dilated, resulting in a residual gradient of 1–2 mmHg. The LUPV showed mild mid-vessel narrowing with a gradient of 5 mmHg, reduced to 2 mmHg after BD. The LLPV was unobstructed. At the age of 11.6 years, progressive disease in the setting of decreased exercise tolerance and worsening right lung perfusion required right-sided anatomic repair surgery including RLPV stent resection, composite right-sided pulmonary vein repair using autologous pericardium and a thin pulmonary homograft patch, augmentation of the lateral pulmonary vein aspect with rotational tissue flaps, atrial septal reconstruction, and right atrial free wall patch plasty. Resected tissue from the RUPV was obtained for downstream analysis for this study. The total follow-up period since anatomic repair surgery was 16.3 months.

The patient was restarted on imatinib mesylate immediately postoperatively (anatomic repair). At two weeks postoperatively, cardiac CT demonstrated unobstructed left pulmonary veins. However, there were some residual stent fragments present near the vein orifices. The RLPV appeared small, and the RUPV was nearly atretic. Since anatomic repair surgery, she has required three catheter-based reinterventions: BD of the RLPV and RUPV at 12.0 years of age (4.2 months postoperatively), and two BDs of the LUPV, RUPV, and RLPV at 12.4 and 13.0 years of age (9.2 and 16.3 months postoperatively). Dapagliflozin propanediol was additionally initiated at 12.4 years of age. Her last catheter-based reintervention demonstrated mildly elevated right-sided filling pressures (RAP 11 mmHg), mildly elevated RV pressures at half systemic levels (RV/systemic pressure ratio 0.52), and mildly to moderately elevated mPAP (26–30 mmHg). The nearly ostial atretic RUPV and RLPV had stenotic segments. Gradients were 10 and 7 mmHg and serially balloon-dilated to 4 and 0–1 mmHg, respectively. The LUPV (previously stented) demonstrated mild recurrent ostial stenosis with a 4 mmHg gradient into the LA. BD reduced the residual gradient to 0–1 mmHg. The LLPV (previously stented) was unobstructed, with a 1 mmHg gradient into the LA. At the most recent follow-up, echocardiography demonstrated an RLPV mean gradient of 5–7 mmHg and an unobstructed LLPV. The upper pulmonary veins were not well visualized. There were signs of mildly elevated RV systolic pressure. The patient continued imatinib mesylate therapy.

### 3.2. Elevated WSS Estimates

Maximum WSS estimates (corresponding to minor diameter) were serially assessed prior to anatomic repair surgery. In patient 1, maximum WSS estimates were markedly elevated in the affected LLPV, RUPV, and RLPV and progressively increased over time, reaching 29.0 (SD: 1.0), 63.33 (SD: 28.87), and 143.3 (SD: 5.77) dyn/cm^2^ at 0.2, 1.8, and 2.7 months of age. Similarly, patient 2 demonstrated pathologically elevated maximum WSS estimates within the affected LUPV and LLPV, which progressively worsened throughout the clinical course. His estimates increased from 28.50 (SD: 14.85) at 0.4 months to 59.50 (SD: 19.09) at 2.3 months and 123.5 (SD: 94.05) dyn/cm^2^ at 10.6 months of age. In patient 3, maximum WSS estimates within the affected LUPV, LLPV, RUPV, and RLPV were also elevated. Her estimates were 25.25 (SD: 23.60), 80.60 (SD: 129.50), and 32.75 (SD: 27.02) dyn/cm^2^ at 9.6, 11.3, and 11.6 years of age.

### 3.3. Tissue Composition Reveals the Underlying Dynamic Fibrotic Process

**Figure 1:**
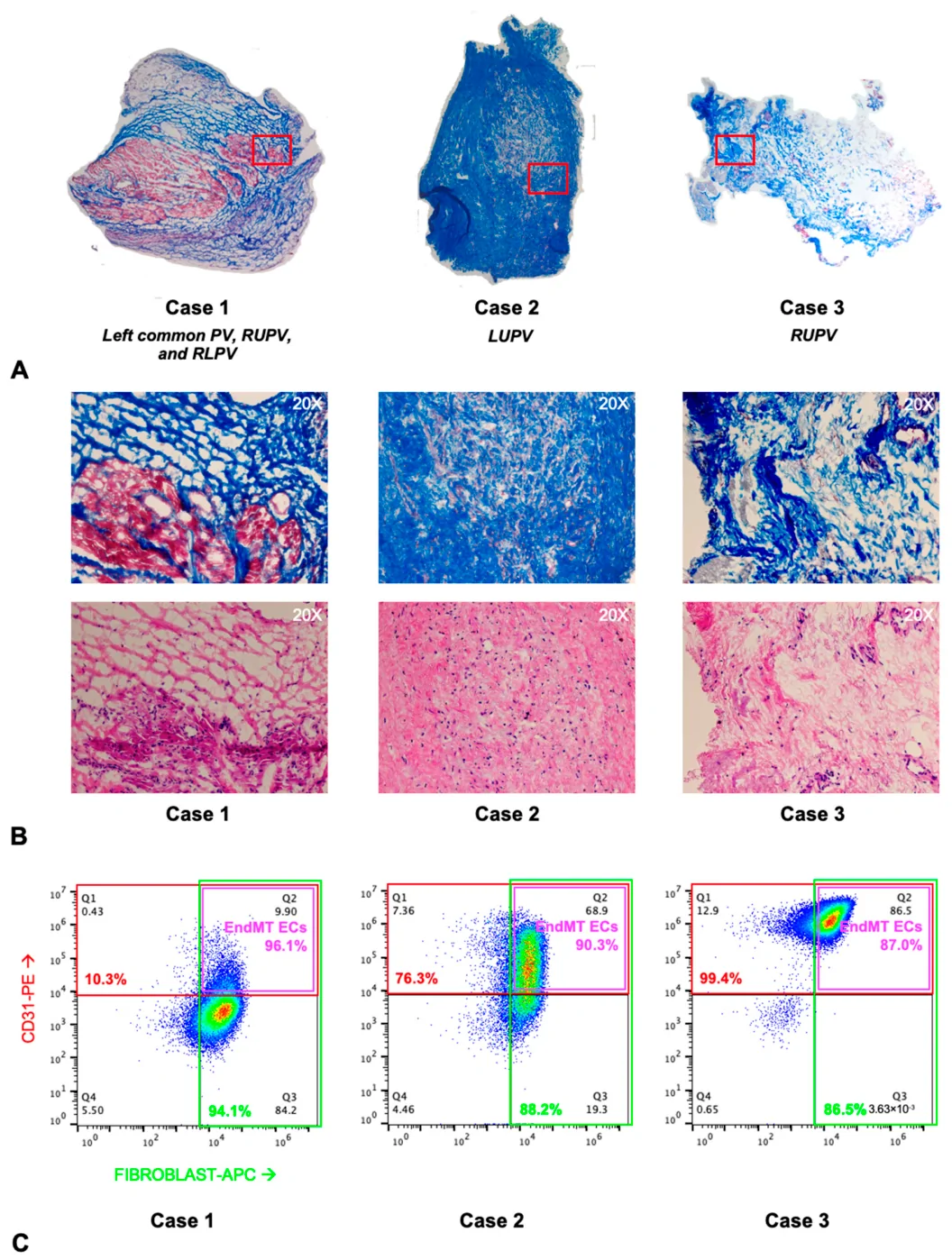
PVS is characterized by fibroproliferative remodeling through endothelial-to-mesenchymal transition of endothelial cells. (**A**) Light microscopy images of Masson’s trichrome (MT)-stained resected whole tissue sections, visualizing the entire specimen, including collagen fibers (blue) and the underlying myocardial sleeve (red). Red boxes mark the regions displayed at higher magnification in (**B**). (**B**) Representative 20× light microscopy images of MT staining (**upper** panel) demonstrating extensive fibrosis, and hematoxylin and eosin (H&E) staining (**lower** panel) highlighting tissue composition, including nuclei (blue) and cytoplasm/extracellular matrix (pink/red). Resected tissue demonstrates abundant organized fibrotic fibers with low cellular density. (**C**) Representative flow cytometry plots showing cells isolated from each resected tissue sample, showing endothelial (CD31) and mesenchymal (fibroblast) marker expression. The majority of cells display a mixed endothelial–mesenchymal phenotype, indicating endothelial cells undergoing fibrotic transition through EndMT as the cellular substrate underlying PVS. CD31-positive cell populations (red boxes); fibroblast-positive cell populations (green boxes); ECs undergoing EndMT (pink boxes).

Macroscopic analysis of the resected tissue revealed a whitish, markedly thickened tissue composition with a minor underlying myocardial sleeve component. Interestingly, MT staining revealed a thick, organized, collagen-positive layer within the lumen of the resected tissue, suggestive of disproportionate fibrosis as a possible underlying pathological process in the pulmonary vasculature (Figure 1). These findings prompted further investigation of respective marker expressions. Flow cytometry of enzymatically digested tissue revealed different degrees of endothelial marker-positive cells. Patient 1 showed very low endothelial marker expression, approximately 10.3%, in his resected tissue. Patients 2 and 3 had comparatively higher yields (76.3% and 99.4%, respectively). In contrast, all patient samples demonstrated a high proportion of fibroblast marker-positive cells, with >85% positive cells detected. Interestingly, all patients also showed a substantial double-positive population, suggesting a dynamic EC transition undergoing EndMT and acquisition of a mesenchymal phenotype. Respective flow cytometry plots are shown in Figure 1.

### 3.4. Cell Culture Experiments

In light of favorable clinical outcomes, we conducted in vitro experiments with both drugs in HECs and in CD144-magnetically isolated PVS-ECs derived from these patients to investigate potential mechanisms underlying the improved clinical course observed in this population.

### 3.5. Imatinib Mesylate and Sirolimus Reduce Cell Viability

**Figure 2:**
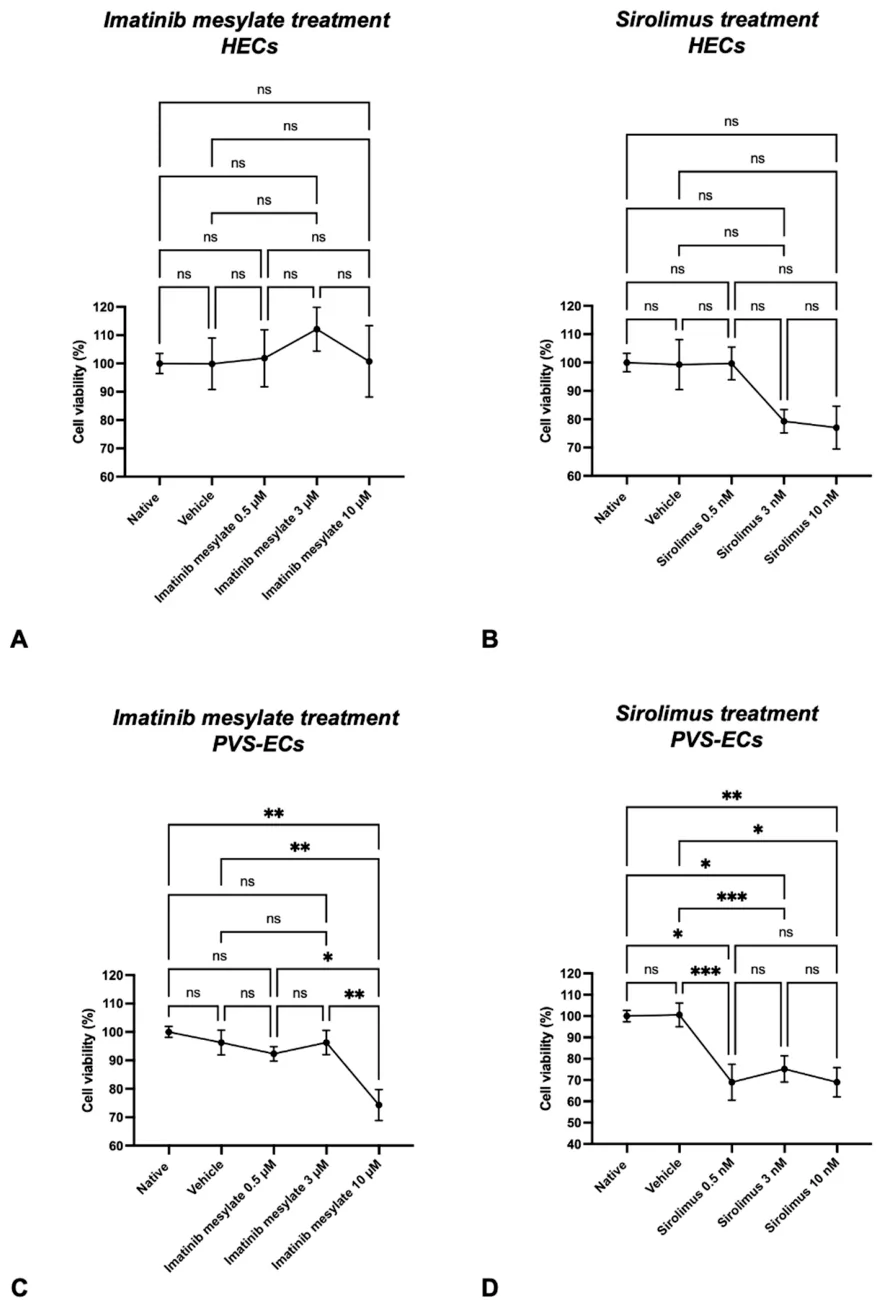
Reduction in cell viability assessed by MTT assay. In HECs (**A**,**B**), neither imatinib mesylate (**A**) nor sirolimus (**B**) significantly affected cell viability compared with untreated controls. Ordinary one-way ANOVA with Tukey’s multiple-comparisons test. In PVS-ECs (**C**,**D**), imatinib mesylate (**C**) significantly decreased cell viability at the highest concentration (10 µM), with a dose-dependent effect. Sirolimus (**D**) significantly decreased cell viability at the lowest concentration tested (0.5 nM). There were no significant differences between the individual treatment concentrations. Repeated-measures one-way ANOVA with Tukey’s multiple-comparisons test. ns, not significant; * *p* < 0.05, ** *p* < 0.01, *** *p* < 0.001.

We performed an MTT assay to evaluate overall drug safety of increasing concentrations of imatinib mesylate and sirolimus. Tested concentrations corresponded to plasma levels with pediatric dosage. In HECs, imatinib mesylate did not reduce cell viability compared to untreated controls (*p* = 0.89). For sirolimus-treated HECs, the overall one-way ANOVA was significant (*p* = 0.009). However, there was no difference in pairwise comparisons after correction for multiple testing. In contrast, the highest concentration of 10 µM imatinib mesylate in PVS-ECs significantly reduced viable cells compared to untreated controls (*p* < 0.0001). This corresponds to peak plasma levels. There was a significant dose-dependent decrease in cell viability from 0.5 µM to 10 µM (*p* = 0.01) and from 3 µM to 10 µM (*p* = 0.004). Sirolimus induced a significant reduction in cell viability in PVS-ECs already at the lowest concentration of 0.5 nM (*p* = 0.0001). No significant differences were observed across the increasing concentrations of sirolimus. Likewise, there were no differences between no treatment and vehicle control conditions. Summary plots of the dose–response experiments are presented in Figure 2. The mean cell viability percentages for each group are summarized in Table A1.

**Figure 3:**
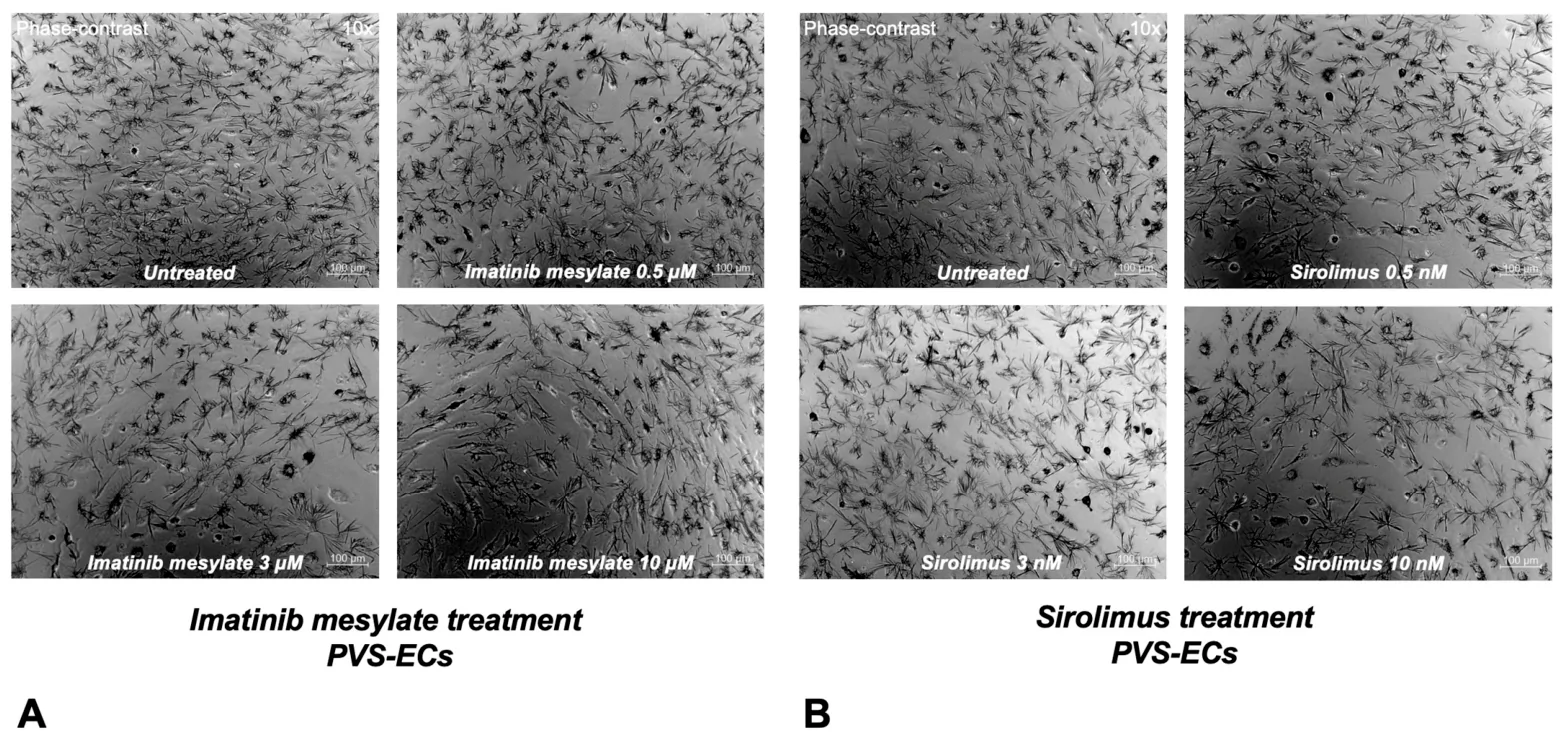
Representative phase-contrast microscopy images of MTT assay in PVS-ECs. PVS-ECs treated for 72 h with imatinib mesylate (**A**) or sirolimus (**B**), and with 3.5 h of MTT incubation, did not exhibit increased numbers of floating cells indicative of cell death and continued to form formazan crystals across increasing treatment concentrations.

Interestingly, phase-contrast microscopy of treated wells after 72 h of drug exposure and 3.5 h of MTT incubation demonstrated that cells in the treatment conditions remained adherent to the wells and continued to form formazan crystals, rather than detaching and floating as expected in cell death (Figure 3). These observations supported our assumption, based on clinical observation, that the reduced number of cells forming formazan crystals, indicative of viable metabolically active cells, was not primarily attributable to increased cytotoxicity.

### 3.6. Imatinib Mesylate and Sirolimus Are Safe to Use for ECs

**Figure 4:**
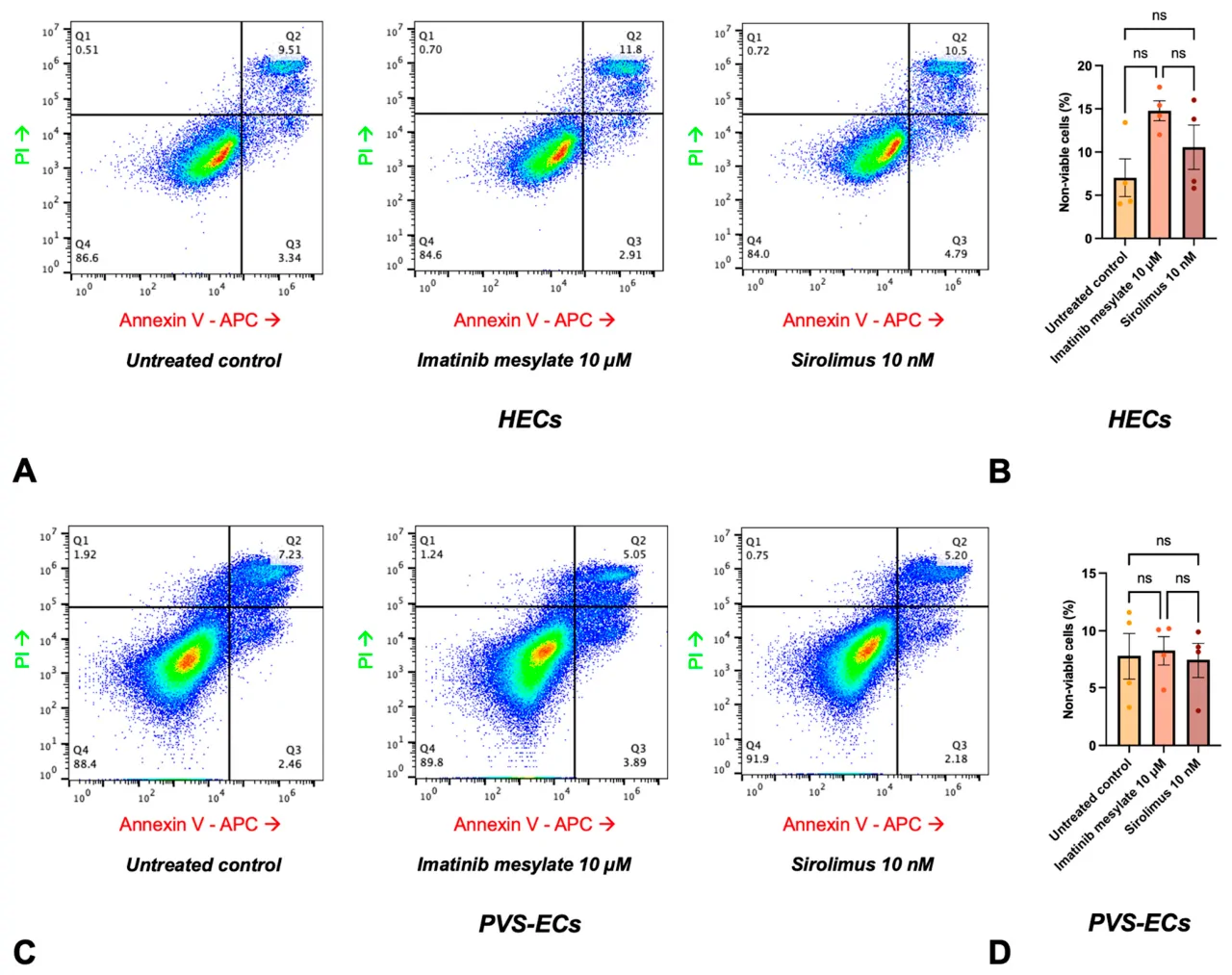
Cell toxicity assessed by Annexin V/PI apoptosis assay. In HECs (**A**,**B**), neither imatinib mesylate nor sirolimus significantly increased apoptotic or dead cells compared with untreated controls. (**A**) Representative flow cytometry plots and (**B**) summary graphs following treatment with imatinib mesylate or sirolimus in HECs. Ordinary one-way ANOVA with Tukey’s multiple-comparisons test. NS, not significant. In PVS-ECs (**C**,**D**), similar findings were observed, with neither imatinib mesylate nor sirolimus significantly increasing apoptotic or dead cells compared with untreated controls. (**C**) Representative flow cytometry plots and (**D**) summary graphs following treatment with imatinib mesylate or sirolimus in PVS-ECs. Repeated-measures one-way ANOVA with Tukey’s multiple-comparisons test. ns, not significant.

To further investigate the underlying mechanism of the observed decrease in cell viability following treatment with imatinib mesylate and sirolimus, we performed an Annexin V/PI apoptosis assay. Based on clinical observations and phase-contrast microscopy of treated cells, we did not anticipate significant toxicity at the concentrations investigated. We therefore examined whether the detected reduction in viable cells was associated with induction of apoptotic cell death. As a representative, we selected the highest concentrations tested of imatinib mesylate (10 µM) and sirolimus (10 nM) to evaluate potential induction of cell death. The treatment with the highest concentrations of imatinib mesylate and sirolimus did not significantly increase apoptotic or dead cells compared with untreated controls in either HECs (*p* = 0.07) or PVS-ECs (*p* = 0.69). Representative flow cytometry plots and summary graphs are shown in Figure 4. Interestingly, although both treatment conditions significantly reduced viable cell numbers after 72 h in PVS-ECs (MTT), neither treatment increased apoptosis, suggesting that the decreased cell viability shown in the MTT assay was not attributable to increased cytotoxicity.

### 3.7. Imatinib Mesylate and Sirolimus Have Anti-Proliferative Characteristics

**Figure 5:**
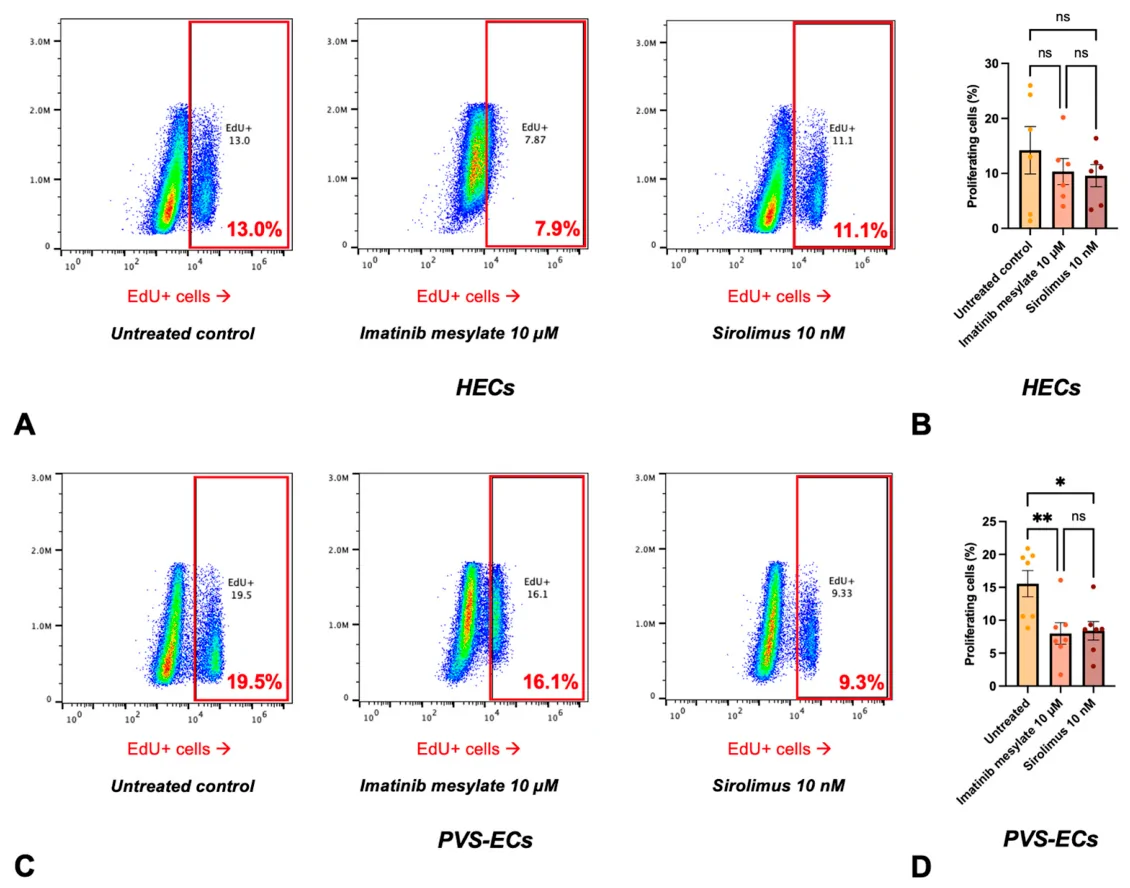
Cell proliferation assessed by EdU proliferation assay. In HECs (**A**,**B**), neither imatinib mesylate nor sirolimus significantly affected cell proliferation compared with untreated controls. (**A**) Representative flow cytometry plots and (**B**) summary graphs following treatment with imatinib mesylate or sirolimus in HECs. Ordinary one-way ANOVA with Tukey’s multiple-comparisons test. NS, not significant. In PVS-ECs (**C**,**D**), both imatinib mesylate and sirolimus significantly reduced cell proliferation compared with untreated controls. No significant difference was observed between imatinib mesylate and sirolimus treatment groups. (**C**) Representative flow cytometry plots and (**D**) summary graphs following treatment with imatinib mesylate or sirolimus in PVS-ECs. Repeated-measures one-way ANOVA with Tukey’s multiple-comparisons test. Red boxes highlight EdU-positive cell populations. ns, not significant; * *p* < 0.05, ** *p* < 0.01.

As imatinib mesylate and sirolimus did not induce apoptosis, we hypothesized that the observed reduction in cell viability was instead related to effects on cellular proliferation. We performed an EdU proliferation assay to detect and quantify newly synthesized DNA through incorporating EdU during treatment. As a representative, the highest concentrations of imatinib mesylate (10 µM) and sirolimus (10 nM) were tested. After 72 h, the EdU proliferation assay in HECs showed that imatinib mesylate and sirolimus did not significantly affect proliferation compared with untreated controls (*p* = 0.54). On the contrary, both treatments significantly decreased cellular proliferation in PVS-ECs (*p* = 0.002). There was no difference between imatinib mesylate and sirolimus in the effects on proliferation observed in either HECs or PVS-ECs. Representative flow cytometry plots and summary graphs are shown in Figure 5.

### 3.8. Imatinib Mesylate and Sirolimus Inhibit EndMT

**Figure 6:**
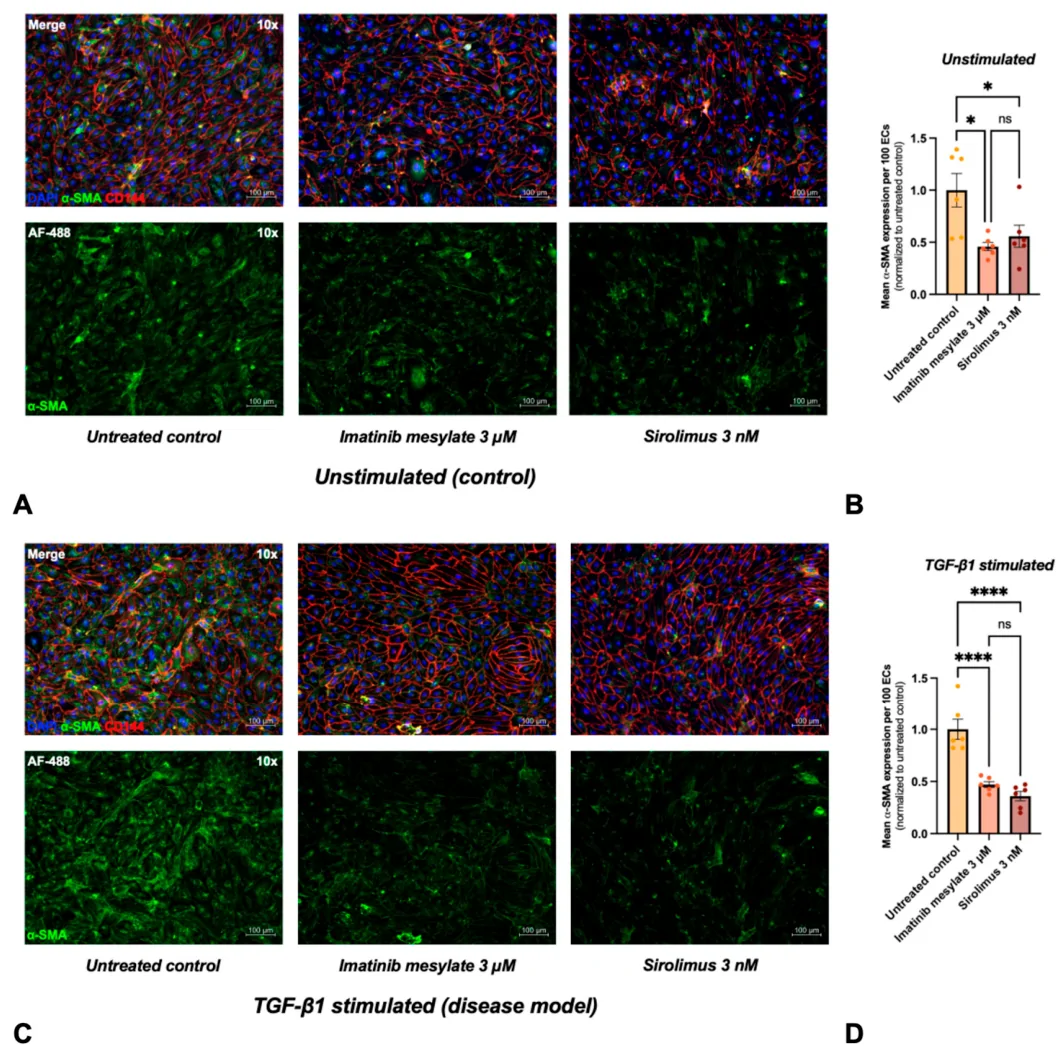
Imatinib mesylate and sirolimus attenuate EndMT. In unstimulated HECs (**A**,**B**), both imatinib mesylate and sirolimus significantly altered EndMT compared with untreated controls, by decreasing the expression of the mesenchymal fibrotic marker α-SMA. (**A**) Representative immunocytochemical images and (**B**) summary graphs following treatment with imatinib mesylate or sirolimus in unstimulated HECs. Ordinary one-way ANOVA with Tukey’s multiple-comparisons test. ns, not significant; * *p* < 0.05. In TGFβ1-stimulated HECs, representing the disease model (**C**,**D**), both imatinib mesylate and sirolimus also significantly reduced EndMT by decreasing α-SMA expression compared with untreated controls. No significant difference was observed between imatinib mesylate and sirolimus treatment groups. (**C**) Representative immunocytochemical images and (**D**) summary graphs following treatment with imatinib mesylate or sirolimus in TGFβ1-stimulated HECs. Repeated-measures one-way ANOVA with Tukey’s multiple-comparisons test. ns, not significant; **** *p* < 0.0001. Fibrotic mesenchymal marker α-SMA (green), endothelial marker CD144 (red), and nuclear marker DAPI (blue).

Because tissue composition in this case series showed extensive fibrotic remodeling and ECs transitioning toward a mesenchymal-like phenotype, we suspected EndMT as a potential signaling pathway targeted by imatinib mesylate and sirolimus. In unstimulated HECs (native), both drugs significantly downregulated the mean expression of the mesenchymal marker alpha-smooth muscle actin (α-SMA) per 100 CD144-positive ECs. Imatinib mesylate induced a 0.46-fold and sirolimus a 0.56-fold downregulation compared to control levels (*p* < 0.01). This indicated decreased EndMT in ECs. In TGF-β1-stimulated HECs, a disease model that mimics profibrotic signaling and induces EndMT, both imatinib mesylate and sirolimus significantly decreased mean α-SMA expression per 100 CD144-positive ECs. Imatinib mesylate and sirolimus induced 0.47-fold and 0.36-fold reductions, respectively (*p* < 0.0001), suggesting a beneficial modulatory effect on ECs undergoing EndMT (Figure 6). There was no difference between imatinib mesylate and sirolimus treatment.

## 4. Discussion

In this study, we have shown that patients with PVS likely exhibited pathologically elevated WSS and vascular fibroproliferative remodeling. This was characterized by pathologically activated ECs that transition into mesenchymal cells through EndMT. Furthermore, we have demonstrated that imatinib mesylate and sirolimus were safe and effective in attenuating vascular fibroproliferative remodeling by inhibiting fibroproliferation and EndMT in PVS-derived ECs.

In this study, all patients were born with PAPVR or TAPVR, both conditions reflecting complex underlying cardiac anatomy that can make surgical management challenging, especially when achieving an entry site free of geometric distortion [25]. Patients 1 and 2 developed PVS post-repair. Patient 3 was born with right-sided PAPVR and left-sided PVS but also developed right-sided PVS post-repair. Initial surgical repair strategies in these patients were more conventional [26,27] and included enlargement of the venous confluence, baffling of the pulmonary veins, unroofing procedures, and/or intraoperative stenting. These, along with preoperative PVO, have been identified as risk factors for postoperative PVS [28]. Surgical interventions may create new hemodynamic conditions. The morphology, especially the pulmonary confluence width at the venous entry site after repair, is associated with the development of postoperative PVS and recurrent stenosis [25]. However, the relative influence of flow turbulence versus pathological flow redistribution remains unclear. We obtained tissue specimens from these patients for downstream analysis when they underwent anatomic repair surgery with augmentation of the pulmonary vein–left atrial connection [26,27], which differed from the approach used in previous surgical interventions. Anatomic repair surgery aims to restore a more physiological pulmonary venous flow profile and, with physiological vessel growth, may also create favorable baseline conditions for more effective dilation of individual veins during subsequent catheter-based reinterventions [26,27,29]. This underscores the value of straightening the pulmonary venous course and minimizing elevated and/or turbulent venous flow. Therefore, the advantageous outcomes observed in these three patients might also be influenced by more physiological hemodynamics following anatomic repair. Nevertheless, in the three patients presented here, PVS persisted or recurred. One potential mechanism may be sustained compression or distortion of the pulmonary veins by surrounding structures as they enter the LA, resulting in suboptimal hemodynamics. While the exact WSS levels that trigger fibroproliferative remodeling in pulmonary veins remain unknown, a recent study suggested that physiological venous WSS levels are below 10 dyn/cm^2^, whereas elevated WSS levels ranging from 20–50 dyn/cm^2^ are associated with vascular remodeling and neointimal hyperplasia [15]. Importantly, not only does the degree of WSS matter, but also whether the flow is laminar or disturbed [14]. All three patients had WSS estimates within or substantially above these reported ranges, supporting the hypothesis that WSS may contribute to PVS onset and progression. However, further mechanistic studies investigating the effects of pathological hemodynamics on EndMT are warranted. From a multifactorial perspective, other less modifiable factors likely contribute to disease susceptibility, including prematurity, genetic predisposition, and epigenetic modulation [30,31].

Notably, some patients develop PVS solely due to localized anatomic obstruction from external compression. Others show extensive neointimal proliferation that drives progressive obstruction of previously unaffected veins and disease recurrence. This neointimal proliferation appears to be the characteristic disease substrate driving this severe and progressive trajectory. Detailed analysis of neointimal proliferation in resected PVS tissue revealed an EC population shifting its marker expression from an endothelial toward a mesenchymal-like phenotype. This is indicative of EndMT. As the innermost vascular layer, ECs are directly exposed to fluid dynamics. EndMT describes a physiological process during cardiac development and tissue homeostasis: the phenotypic and functional transition of ECs into mesenchymal-like cells [32,33]. However, this process can be pathologically reactivated in response to stimuli such as hyperglycemia [34], inflammation [35], hypoxia [36], and, as our group and others have previously shown, abnormal mechanical strain [37] and disturbed hemodynamics [14,23,38]. The importance of the latter is further pointed out by a recent study using RNA sequencing, which found that PIEZO1, a mechanosensitive receptor expressed in the endothelium, is a potential mediator of PVS [39].

A key signaling pathway underlying EndMT is the SMAD/TGF-β1 pathway, which can be activated by mechanical stimuli such as altered hemodynamics. Downstream-activated transcription factors, such as Slug/Snail and TWIST, drive the downregulation of endothelial genes, including PECAM-1 and VE-cadherin, and the upregulation of mesenchymal markers such as vimentin and α-SMA, therefore promoting a transition from an endothelial to a mesenchymal-like phenotype. Patient 1, with the highest WSS estimates, showed very limited expression of endothelial markers (10.3%). Patient 2, with WSS estimates intermediate between those of patients 1 and 3, showed an intermediate degree of endothelial marker expression (76.3%). Patient 3, with the lowest, yet still pathological, WSS estimates, demonstrated the highest yield of endothelial marker-positive cells (99.4%). All patients had a high content of fibroblast-positive cells (86.5–94.1%). Overall, endothelial marker expression appeared to correlate with WSS estimates, suggesting an association between pathologically elevated WSS in the pulmonary vein lumen and ECs undergoing EndMT. Restoring an adequate lumen diameter in a stenotic vein may create favorable hemodynamics and outcomes [15,25,40]. Attenuating pathological mechanical stimuli that trigger EndMT may thereby reduce fibroproliferative remodeling and recurrent stenosis. In all patients, transitioning double-positive ECs expressing both endothelial and mesenchymal markers were present, indicating that PVS is still a dynamic, ongoing process even at advanced disease stages. This process is likely targetable, as we have previously demonstrated in other entities with losartan [41] and atorvastatin [24].

In our cell culture studies, we observed differences in cell viability between HECs and PVS-ECs as measured by the MTT assay. Interestingly, imatinib mesylate and sirolimus were well tolerated in HECs, whereas both significantly reduced cell viability in PVS-ECs. Importantly, HECs are slowly proliferating cells that typically become quiescent at confluence. Mesenchymal-like cells, especially when activated, proliferate rapidly. Although both drugs have been reported to induce apoptosis, most previous studies used substantially higher concentrations or were conducted in cancer cell lines. Our in vitro results show that imatinib mesylate and sirolimus are well tolerated in ECs. We tested sirolimus concentrations far below this range, which corresponded to clinically relevant dosing levels of 1 mg/m^2^ once daily and target serum concentrations of 8–12 ng/mL (approximately 8.8–13.1 nM). From a clinical perspective, as others have shown, sirolimus treatment is safe and reliable, and toxicity is typically dose-dependent and most commonly occurs when drug levels exceed 15 ng/mL [42]. Imatinib mesylate is primarily used in cancer therapy for leukemia, gastrointestinal stromal tumors, and myeloproliferative diseases. In pediatric patients, the dosage is 340 mg/m^2^ once daily, which results in target serum concentrations of 800–3000 ng/mL (approximately 1.6–6.1 µM). Therefore, at concentrations tested in this study, the effects were predominantly antiproliferative. In PVS-ECs, fibroproliferation implies a promising therapeutic target. Recently, the first experimental evidence with a cellular correlate has demonstrated that PVS is a true fibroproliferative disease by showing increased proliferation of mesenchymal cells in a neonatal rodent model of PVS [43]. Sirolimus has reduced neointimal proliferation in this model, consistent with our findings. However, the cellular origin of this intraluminal mesenchymal proliferation and wall thickening has remained unclear. Through analyzing the cellular composition of resected human tissue, we identified ECs undergoing EndMT toward a mesenchymal phenotype as the likely cellular driver, supporting a targeted anti-fibroproliferative therapy. Consistent with published rodent data and current clinical hypotheses, imatinib mesylate and sirolimus reduced proliferation in PVS-ECs. As with our MTT results, we did not detect reduced proliferation in HECs.

In addition to targeting fibroproliferation, we demonstrated that imatinib mesylate and sirolimus inhibit vascular remodeling by suppressing EndMT. Platelet-derived growth factor receptor beta (PDGFR-β), a receptor tyrosine kinase highly expressed in activated stromal cells, has also been shown to be highly expressed in PVS [44]. Imatinib mesylate is a tyrosine kinase inhibitor that also inhibits PDGFR-β signaling. Interestingly, pharmacologic PDGF receptor inhibition has been shown to alleviate long-term cardiac remodeling and fibrosis [45]. Sirolimus effectively inhibits the mammalian target of rapamycin (mTOR), which has also been recently shown to be activated in an animal model of PVS [46]. Using new, precise drug delivery systems in PVS might specifically target these diseased activated cell states [47]. In the context of PVS, it is important to highlight that mTOR inhibitors have recently emerged as promising therapeutic candidates due to their ability to prevent vascular proliferative disease [48]. While we cannot conclude that the observed antifibrotic effects on EndMT in PVS-ECs are mediated by inhibition of PDGFR-β and mTOR signaling pathways, this may be a plausible mechanistic pathway warranting future investigation.

All three patients included in this study required only catheter-based reinterventions. No stent implantation or surgical reintervention was required. Although serial WSS estimate follow-up data are not yet available, follow-up catheterization reports indicate that pressure gradients within the affected pulmonary veins continued to increase over time, but repeated BDs reduced them to an acceptable range. However, the initial protective effect of anatomic repair surgery [26,27], along with repeated catheter-based interventions [29] that increased vein diameter and patency and physiological vein growth, may also have helped to achieve a more physiological flow profile. Considering the severity of the disease, achieving a controlled stage represents a major advancement. However, the continued need for reinterventions indicates that the underlying mechanisms causing PVS remain incompletely understood. However, the pathomechanisms underlying PVS are multifactorial. The underlying disease processes in primary and secondary PVS may differ substantially. Consequently, responses to medical therapies may also vary between these patient populations. All three patients in our cohort had anomalously connected pulmonary veins and developed post-repair PVS. Additional investigation is needed in other populations, such as those with prematurity, or in patients with normally connected veins.

To summarize, this study shows that imatinib mesylate and sirolimus therapy may promote protective vascular fibroproliferative remodeling by inhibiting hyperproliferation and reducing the expression of profibrotic markers in pathologically altered ECs. Even in patients who may be considered clinically burned out, the tissue composition is not exclusively fibrotic, suggesting that active, targetable cellular processes are still ongoing. This provides a cellular rationale for the use of anti-fibroproliferative agents to achieve a stable cellular disease state that may be well controlled with repeated catheter-based reinterventions, thus improving long-term outcomes and survival. Combining surgical and catheter-based interventions with adjunctive pharmacologic therapy may be a promising regimen. A multimodal treatment strategy is likely to improve outcomes in this highly complex and heterogeneous patient cohort.

## 5. Limitations

This study is investigational, so the results should therefore be interpreted carefully. Due to the small sample size, these findings may not be generalizable. Statistically, a type 2 error cannot be excluded. The observed beneficial clinical course should be interpreted in the context of potential confounders. Catheter-based interventions and anatomical repair strategies may independently improve hemodynamics. Potential antifibrotic effects of concomitant medications cannot be excluded. The methodology for WSS estimates does not recapitulate the complex three-dimensional, pulsatile flow dynamics at the pulmonary venous–atrial junction but rather estimates pathological high-shear forces. EC monocultures under static conditions do not recapitulate the complexity of systemic, whole-organism effects. This must be taken into consideration when extrapolating our in vitro findings to the hemodynamic conditions observed in patients. Prospective studies in larger cohorts are necessary to validate our findings. Nevertheless, our study delivers valuable insights into the cellular mechanisms underlying PVS and how they may be effectively targeted.

## 6. Conclusions

Patients with PVS exhibit pathologically elevated WSS and obstructive neointimal hyperplasia. This is characterized by fibroproliferative remodeling driven by ECs undergoing EndMT. These fibrogenically activated ECs represent a promising therapeutic target and may be safely and effectively attenuated by treatment with imatinib mesylate and sirolimus. A multimodal treatment approach may improve clinical outcomes.

## Data Availability

The original contributions presented in this study are included in the article. Further inquiries can be directed to the corresponding author.

## Abbreviations

The following abbreviations are used in this manuscript:

ASD	Atrial septal defect
BD	Balloon dilation
BSA	Bovine serum albumin
CD144	Cluster of differentiation 144
CHD	Congenital heart disease
CT	Computed tomography
DAPI	4′,6-diamidino-2-phenylindole
DMSO	Dimethyl sulfoxide
DNA	Deoxyribonucleic acid
ECs	Endothelial cells
EdU	5-ethynyl-2′-deoxyuridine
EDTA	Ethylenediaminetetraacetic acid
EndMT	Endothelial-to-mesenchymal transition
FACS	Fluorescence-activated cell sorting
HECs	Healthy endothelial cells
LA	Left atrium
LAP	Left atrial pressure
LLPV	Left lower pulmonary vein
LUPV	Left upper pulmonary vein
mPAP	Mean pulmonary arterial pressure
mTOR	Mammalian target of rapamycin
MTT	3-(4,5-dimethylthiazol-2-yl)-2,5-diphenyltetrazolium bromide
NGS	Normal goat serum
PA	Pulmonary artery
PAPVR	Partially anomalous pulmonary venous return
PBS	Phosphate-buffered saline
PDGF-BB	Platelet-derived growth factor BB
PDGFR-β	Platelet-derived growth factor receptor beta
PFO	Patent foramen ovale
PH	Pulmonary hypertension
PI	Propidium iodide
PVO	Pulmonary venous obstruction
PVS	Pulmonary vein stenosis
RAP	Right atrial pressure
RLPV	Right lower pulmonary vein
RMPV	Right middle pulmonary vein
RT	Room temperature
RUPV	Right upper pulmonary vein
RV	Right ventricle
SD	Standard deviation
SEM	Standard error of the mean
TAPVR	Total anomalous pulmonary venous return
TGF-β1	Transforming growth factor beta 1
WSS	Wall shear stress
α-SMA	Alpha smooth muscle actin

## Appendix A

**Table A1:** Mean cell viability following treatment with imatinib mesylate and sirolimus.

HECs; Imatinib mesylate					
Group	Native	Vehicle	0.5 µM	3 µM	10 µM
% (SD)	100 (17.85)	99.85 (45.50)	101.9 (50.37)	112.1 (38.76)	100.7 (62.96)
HECs; Sirolimus					
Group	Native	Vehicle	0.5 nM	3 nM	10 nM
% (SD)	100 (16.44)	99.29 (44.27)	99.68 (28.92)	79.29 (20.59)	77.04 (37.85)
PVS-ECs; Imatinib mesylate					
Group	Native	Vehicle	0.5 µM	3 µM	10 µM
% (SD)	100 (8.71)	96.29 (19.50)	92.33 (11.36)	96.28 (19.11)	74.30 (24.56)
PVS-ECs; Sirolimus					
Group	Native	Vehicle	0.5 nM	3 nM	10 nM
% (SD)	100 (8.42)	100.6 (17.63)	68.98 (26.59)	75.16 (19.42)	68.94 (21.67)
